# Supplementary material for: Gate-tunable giant nonreciprocal charge transport in noncentrosymmetric oxide interfaces
Source: Nat Commun. 2019 Oct 4;10:4510. doi: 10.1038/s41467-019-12466-1 (PMC6778138; doi:10.1038/s41467-019-12466-1)
Supplement: Supplementary file 1 — Supplementary Information [file 41467_2019_12466_MOESM1_ESM.pdf]

Supplementary Information for

Gate-tunable giant nonreciprocal charge transport in noncentrosymmetric oxide interfaces

Choe and Jin *et al.*

Supplementary Information for:

Gate-tunable giant nonreciprocal charge transport in noncentrosymmetric oxide interfaces

Daeseong Choe[1]†, Mi-Jin Jin[1]†, Shin-Ik Kim[2], Hyung-Jin Choi[2], Junhyeon Jo[1], Inseon Oh[1], Jungmin Park[1], Hosub Jin[3], Hyun Cheol Koo[4,5], Byoung-Chul Min[4], Suk-Min Hong[4], Hyun-Woo Lee[6], Seung-Hyub Baek[2,7] & Jung-Woo Yoo[1]*

[1]School of Materials Science and Engineering-Low dimensional Carbon Materials Center, Ulsan National Institute of Science and Technology, Ulsan, 44919, Korea.

[2]Center for Electronic Materials, Korea Institute of Science and Technology, Seoul, 02792, Korea.

[3]Department of Physics, Ulsan National Institute of Science and Technology, Ulsan, 44919, Korea.

[4]Center for Spintronics, Korea Institute of Science and Technology, Seoul, 02792, Korea.

[5]KU-KIST Graduate School of Converging Science and Technology, Korea University, Seoul, 02481, Korea

[6]Department of Physics, Pohang University of Science and Technology, Pohang, 37673, Korea.

[7]Division of Nano & Information Technology, KIST School, Korea University of Science and Technology, Seoul, 02792, Korea

†These authors equally contributed to this work

Correspondence and requests for materials should be addressed to J.-W. Y. (email: jwyoo@unist.ac.kr)

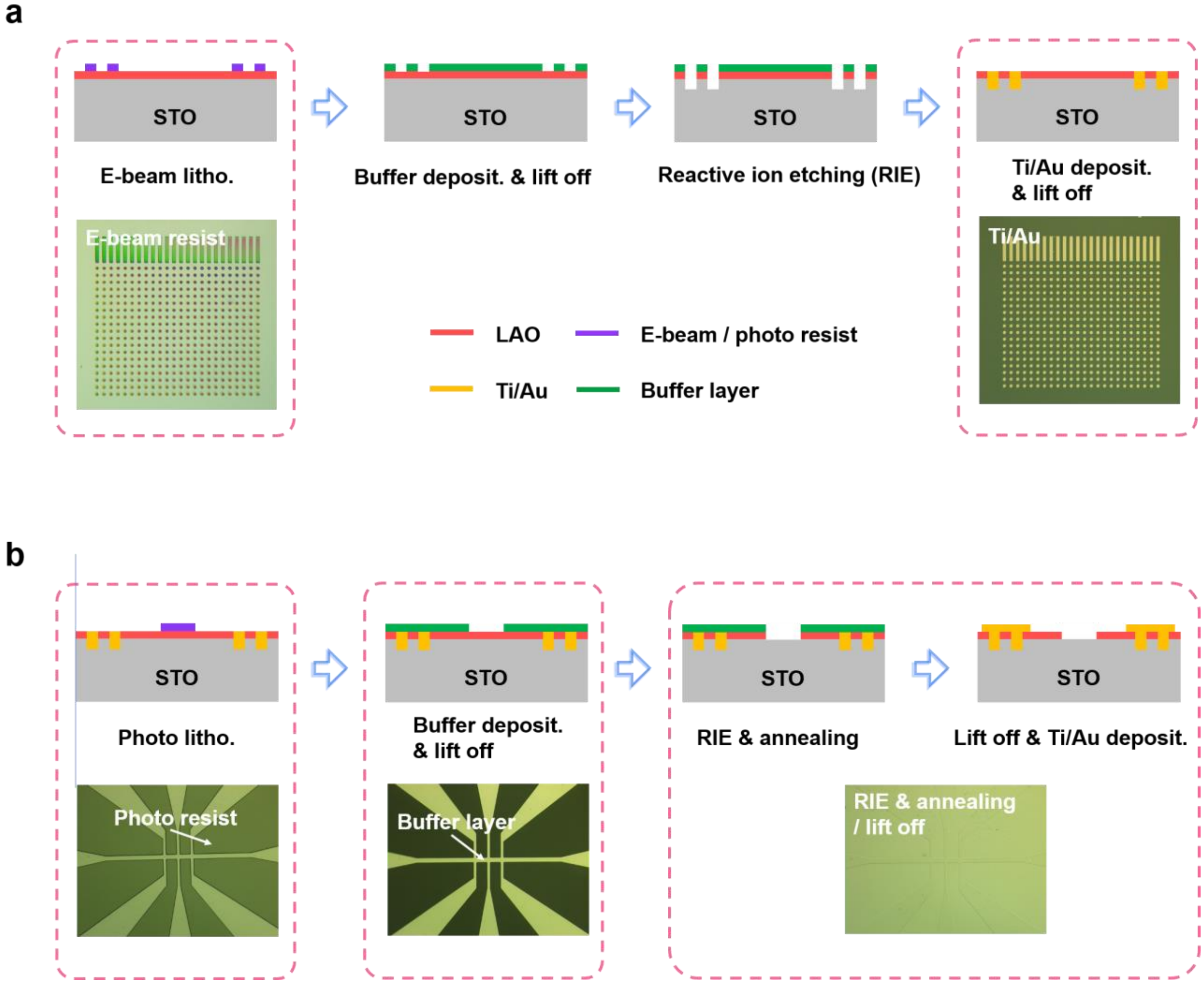

Supplementary Figure 1. Schematic illustration of the device fabrication. a Fabrication procedure for contacts to 2DEG. **b** Fabrication procedure for a Hall bar patterning and contact electrodes. Schematics are side views and optical images are the top views of the device at each fabrication step.

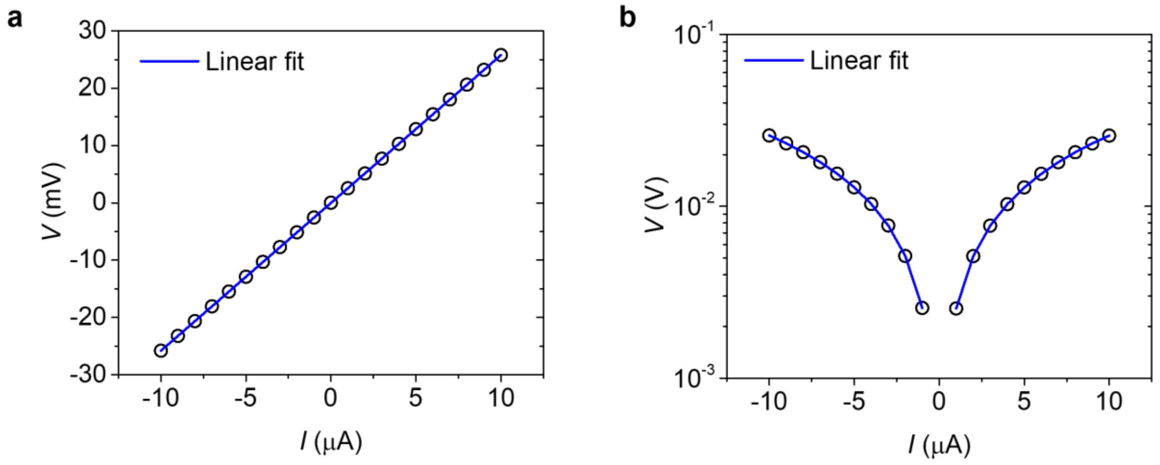

Supplementary Figure 2. Four-terminal *I-V* curve measured for the device B. a A linear *I-V* curve measured at 8 K. **b** A semi-logarithmic plot of the measured *I-V* curve at 8 K. Blue lines are linear fits displaying Ohmic contacts between the conductive interfaces and the Ti/Au electrodes.

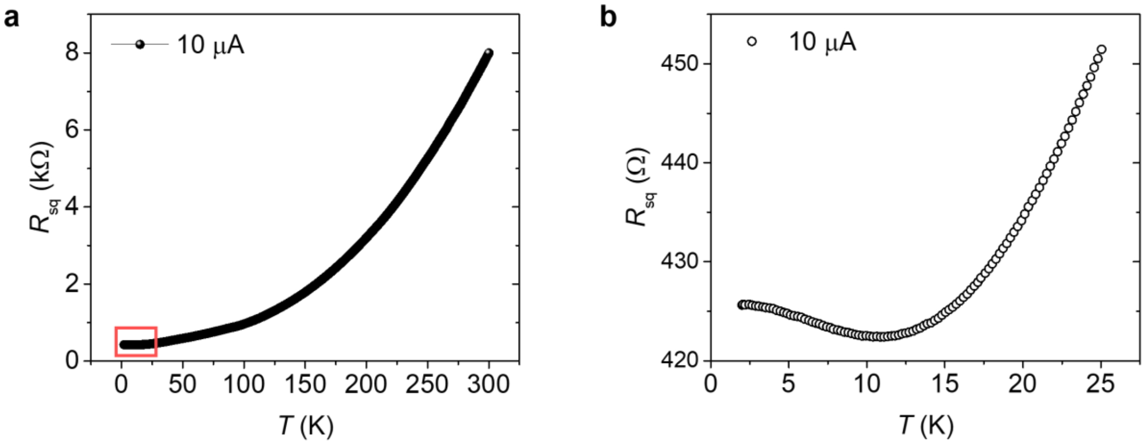

Supplementary Figure 3. Temperature dependence of resistance measured for the device B. **a** Temperature dependence of resistance from 2 to 300 K. **b** Temperature dependence of resistance at low *T* below 25 K. The increase in the measured resistance below ~ 11 K can be attributed to both Kondo effect[1] and weak localization[2].

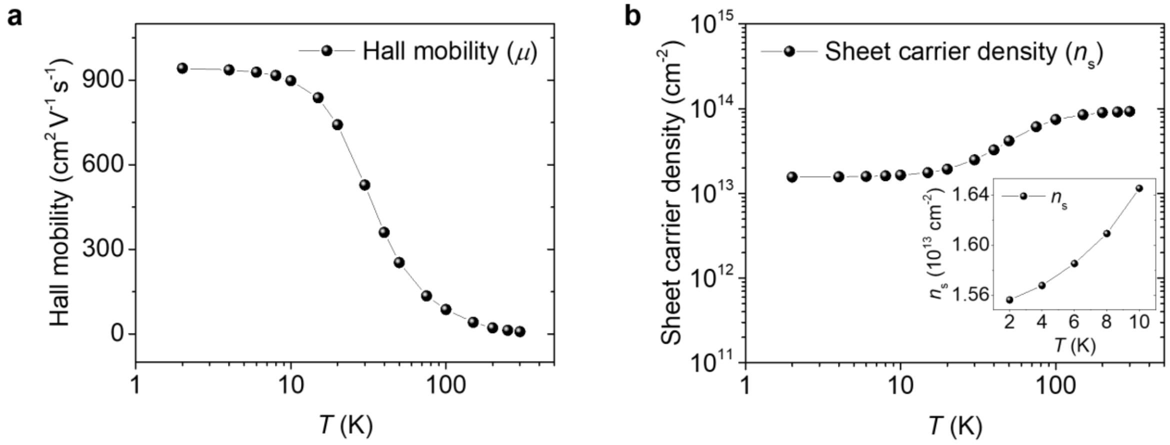

Supplementary Figure 4. Electrical properties measured for the device B. a Temperature dependence of the electron mobility. **b** Temperature dependence of the sheet carrier density estimated from Hall measurements. The inset displays the sheet carrier density from 2 K to 10 K.

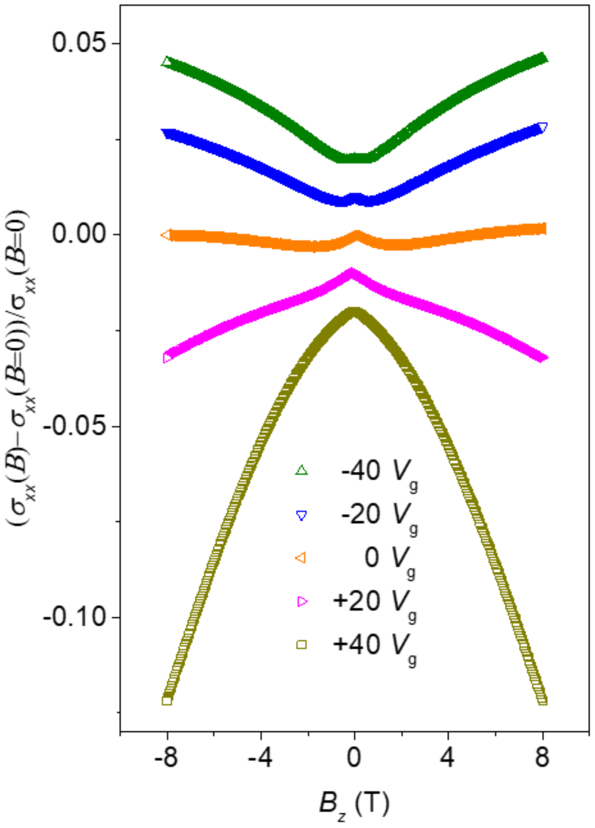

Supplementary Figure 5. Magnetoconductance as a function of the perpendicular magnetic field measured at various V_g. Measurements were done with I = 30 μA at 2 K for the device C. For a negative gate voltage (−40 V_g), a positive magnetoconductance can be observed indicating the dominance of weak localization due to weak spin-orbit interaction. As the applied gate voltage increased, a positive magnetoconductance was gradually turned into a negative magnetoconductance indicating that the charge transport relies on the weak anti-localization. Crossover from weak localization to weak anti-localization is associated with the gate-tunable Rashba spin-orbit interaction in LAO/STO[2].

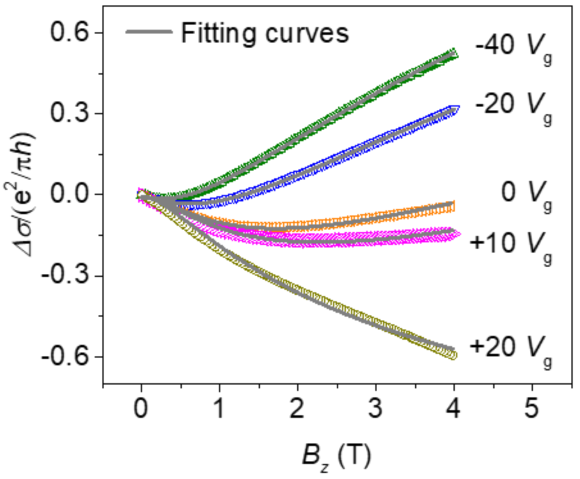

Supplementary Figure 6. Analysis of magnetoconductance based on Maekawa-Fukuyama theory. The magnetoconductance $\Delta\sigma = \sigma(B) - \sigma(B = 0)$ normalized by the quantum of conductance $e^2/\pi h$ at various V_g. Grey lines represent best fits to the measured magnetoconductance using Maekawa-Fukuyama theory. Measurements were done with $I = 30$ μA at 2 K for the device C.

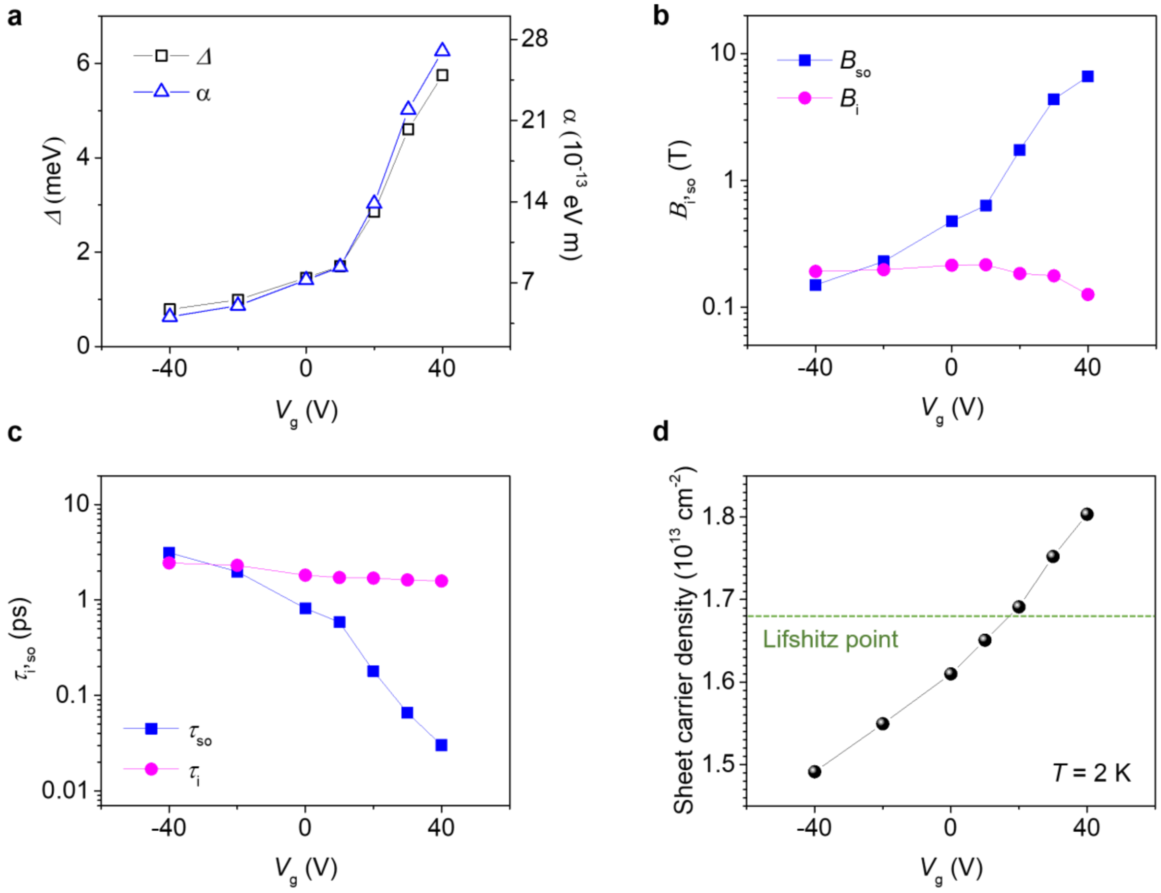

Supplementary Figure 7. Analysis of the Rashba spin-orbit interaction at the $LaAlO_3/SrTiO_3$ interface. a Left axis, black squares: V_g dependence of the Rashba spin splitting Δ. Right axis, blue triangles: V_g dependence of the Rashba spin-orbit interaction constant α. **b** V_g dependence of the fitting parameters B_i (magenta circles) and B_{so} (blue squares). c Inelastic relaxation time τ_i (magenta circles) and spin relaxation time τ_{so} (blue squares) as a function of V_g plotted on a logarithmic time scale. **d** Carrier concentration n_s as a function of V_g.

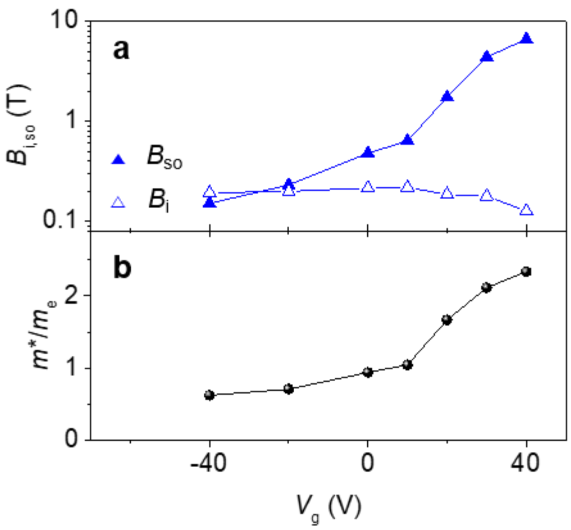

Supplementary Figure 8. The estimated effective mass m^* and spin-orbit field B_{so} as a function of V_g. **a** The obtained fitting parameters, B_i and B_{so}, as a function of V_g. **b** V_g dependence of the effective mass m^*. Here m_e is the bare electron mass.

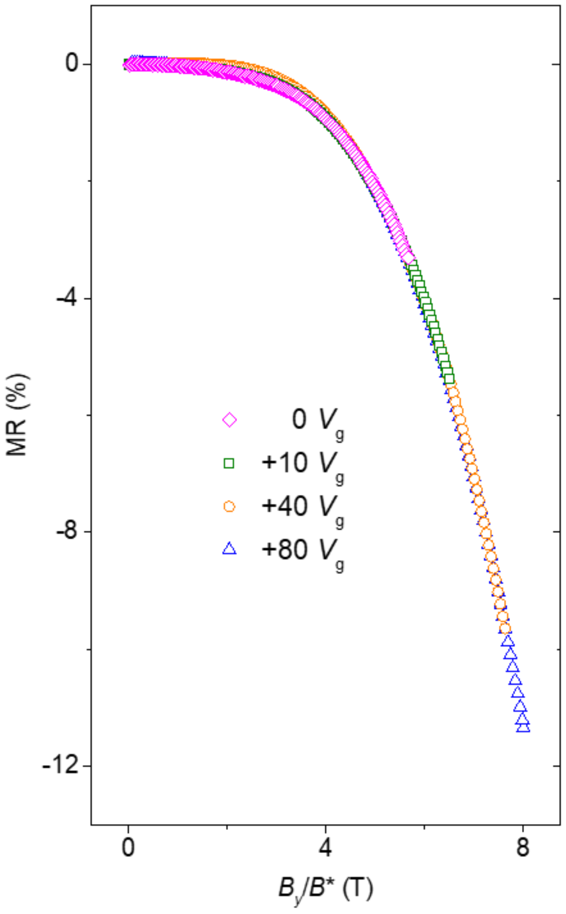

Supplementary Figure 9. Magnetoresistance as a function of a rescaled magnetic field B_y/B^* at different V_g. The magnetoresistance curves at different V_g (shown in Fig. 2a) collapse onto a single curve with a rescaled magnetic field of B_y/B^*, where B^* is a Fermi level dependent value. Measurements were done with $I = 30$ μA at $T = 8$ K.

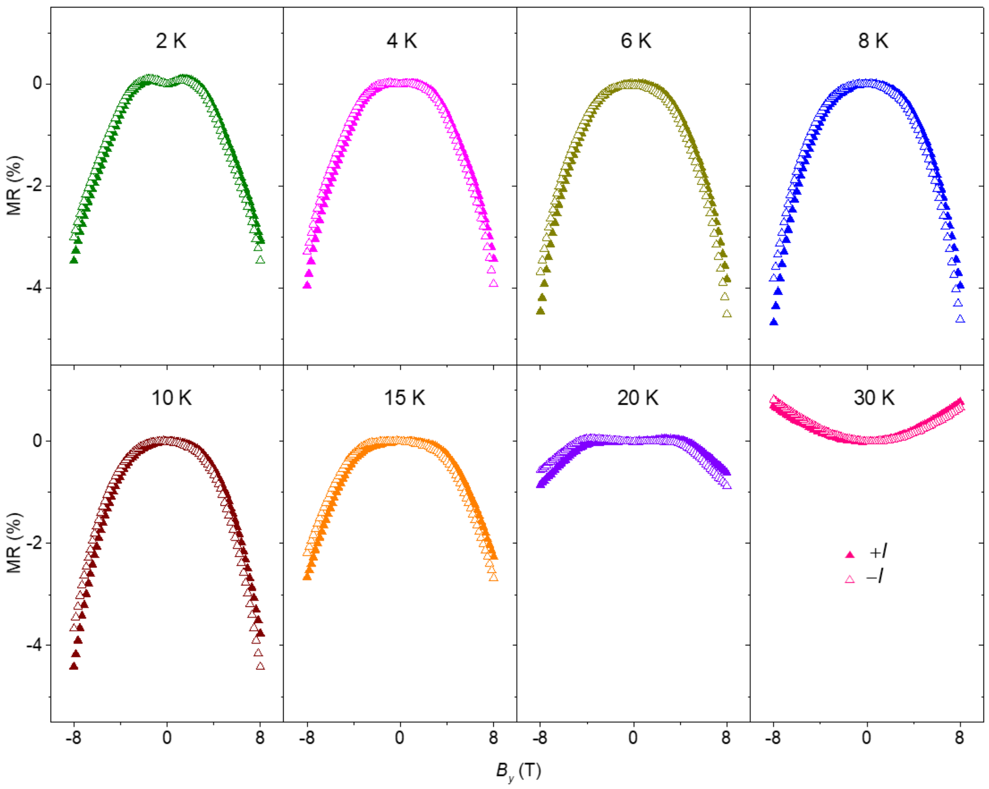

Supplementary Figure 10. In-plane magnetoresistance curves measured at various temperatures. The negative in-plane magnetoresistance persists up to 20 K. Measurements were done with $I = \pm 30$ μA for the device D.

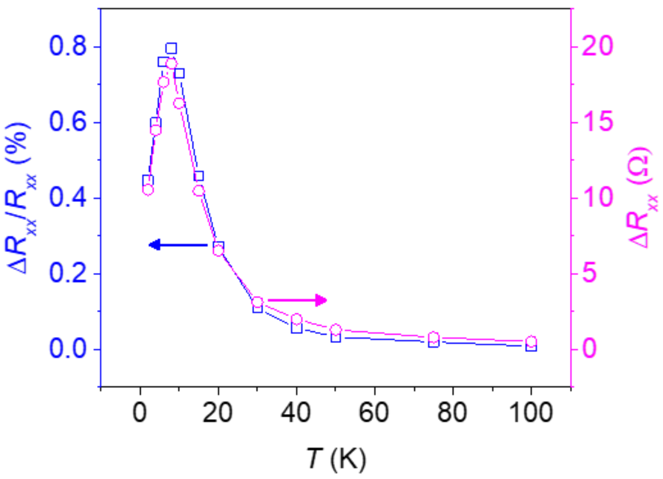

Supplementary Figure 11. The temperature dependence of $\Delta R_{xx}/R_{xx}$ and ΔR_{xx}. The temperature dependence of $\Delta R_{xx}/R_{xx}$ (left axis) and ΔR_{xx} (right axis). Both $\Delta R_{xx}/R_{xx}$ and ΔR_{xx} showed maximum at around 10 K and nearly disappeared over 100 K. Measurements were done with I = 30 μA and B_y = 8 T for the device D. The nonreciprocal responses persist over several tenth Kelvin. We note that the observed ΔR_{xx} is not associated with magnetothermal effect, which appears in the vicinity of the small magnetic field at very low temperature (< ~ 800 mK)[3].

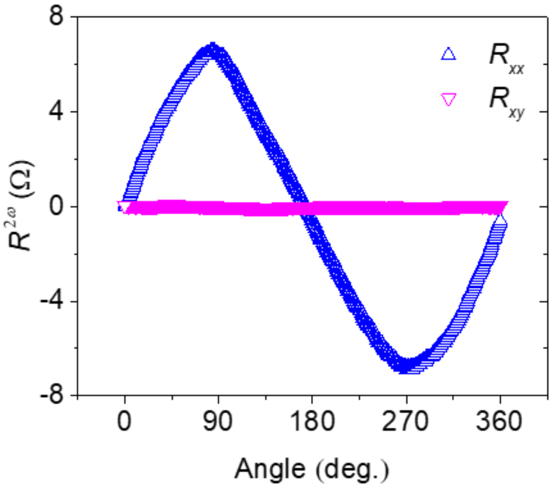

Supplementary Figure 12. Thermoelectric effect on $R^{2\omega}$. $R^{2\omega}$ of R_{xx} and R_{xy} were measured with an applied magnetic field of B = 8 T rotated in the xy plane. Measurements were done with I_{ac} = 105 μA at 2 K (device E). R_{xx} clearly shows sin θ like behavior of $R^{2\omega}$ with J = ~ 1.4 × 10^5 A cm^{-2}. Although the LAO/STO interface exhibits ferromagnetism, $R^{2\omega}$ of R_{xy} does not show cos θ behavior. This indicates absence of the thermoelectric effects, which are associated with a longitudinal spin Seebeck effect, an anomalous Nernst effect, and Nernst effect due to a vertical temperature gradient. In addition, we didn't observe cos θ behavior of $R^{2\omega}$ of R_{xy} when the applied current density was increased up to J = 1.4 × 10^7 A cm^{-2} [4].

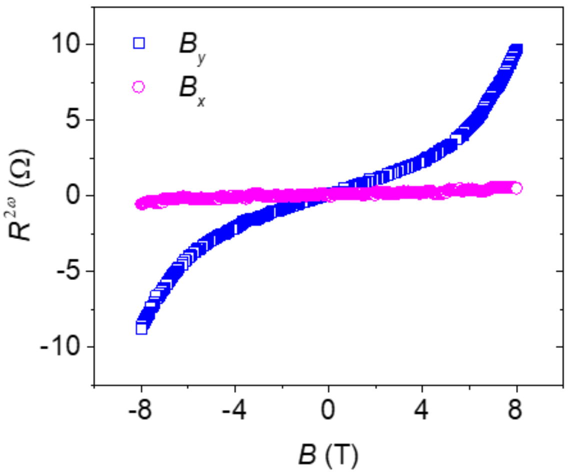

Supplementary Figure 13. **Magnetic field dependence of $R^{2\omega}$.** $R^{2\omega}$ measured for different magnetic field directions B_y and B_x, respectively. Measurements were done for the device B with I_{ac} = 200 μA at 8 K. The nonzero $R^{2\omega}$ signal from the nonreciprocal charge transport appears at the applied magnetic field B_y which is perpendicular to the current, whereas $R^{2\omega}$ is almost zero at the applied magnetic field B_x that is parallel to the current.

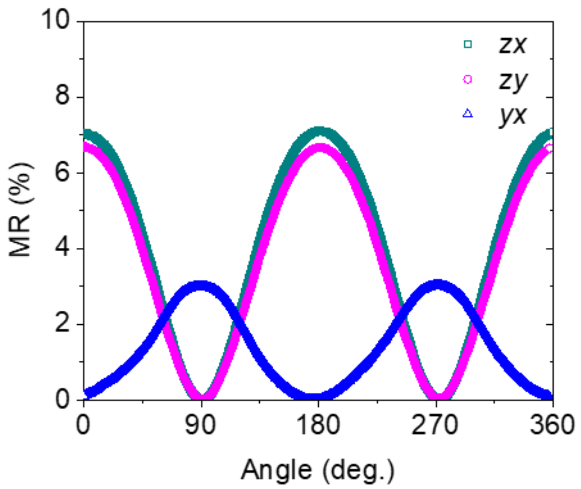

Supplementary Figure 14. Angle dependent magnetoresistance of R^{ω}. R^{ω} was measured with an applied magnetic field of $B = 8$ T rotated in three orthogonal plans (*yx*, *zy*, *zx*). Measurements were done with an applied current of $I_{ac} = 105$ μA at 2 K (device F). The zero angles are at +*y*, +*z*, and +*z* and the directions of rotations are *y* to *x*, *z* to *y*, and *z* to *x* for *yx*, *zy*, *zx* rotations, respectively (see schematic illustration in Fig. 3A). R^{ω} is invariant under reversal of the magnetic field showing a sinusoidal angular dependence with a period of 180°.

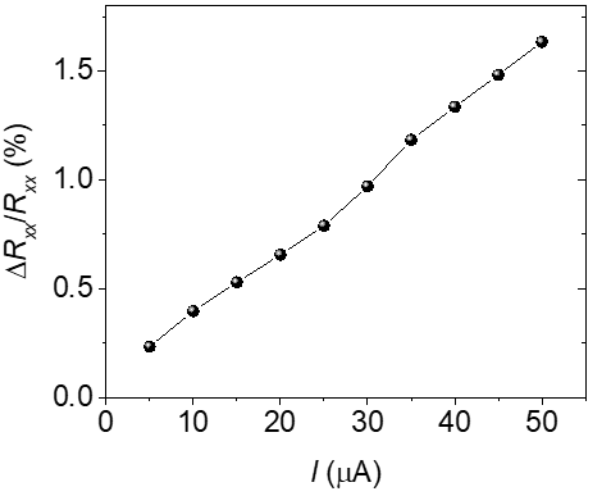

Supplementary Figure 15. The ratio of resistance change ΔR_{xx}/R_{xx} as a function of the applied DC current. Measurements were done for the device A at T = 8 K and B_y = 8 T. Results display that ΔR_{xx}/R_{xx} linearly depends on the applied current I_x.

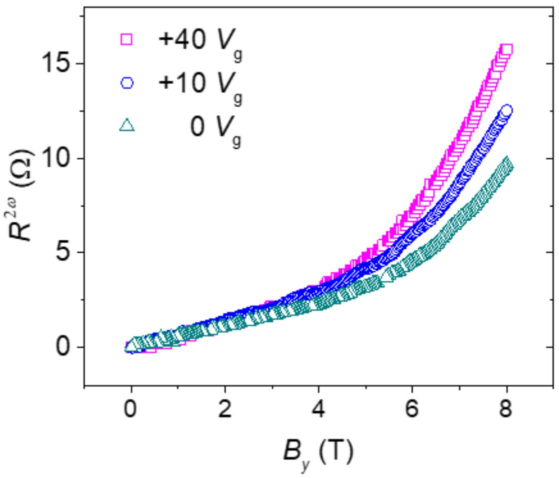

Supplementary Figure 16. $R^{2\omega}$ *vs* B_y measured at different gate voltages (Device B). Measurements were done with fixed gate voltages while sweeping the applied magnetic field B_y at T = 8 K. The applied AC current was I_{ac} = 200 μA.

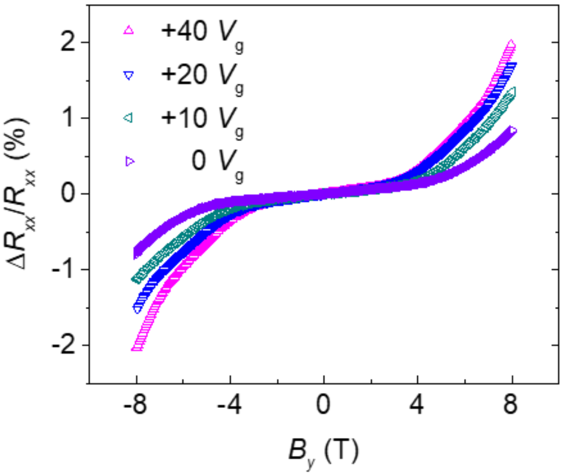

Supplementary Figure 17. $\Delta R_{xx}/R_{xx}$ *vs* B_y measured for an applied DC current at different gate voltages (device A). ΔR_{xx} were estimated by subtracting $R_{xx}(+I)$ from $R_{xx}(-I)$. Each $R_{xx}(\pm I)$ were measured while sweeping the magnetic field at fixed gate voltages at T = 8 K. The applied DC current was I = 30 μA.

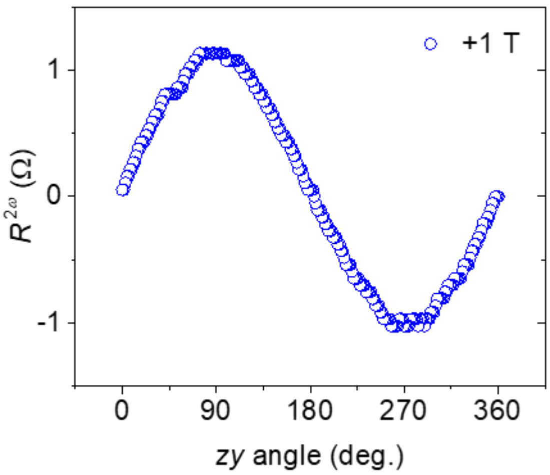

Supplementary Figure 18. $R^{2\omega}$ as a function of the *zy* angle measured with the applied magnetic field of +1 T. The observed $R^{2\omega}$ displayed a sinusoidal angular dependence. Measurements were done at I_{ac} = 200 μA and T = 8 K for the device D.

Supplementary Note 1. Analysis of the magnetoconductance of the $LaAlO_3/SrTiO_3$ interface.

The magnetoconductance as a function of magnetic field was measured at 2 K with various applied V_g (Supplementary Fig. 5). At −40 V_g, the positive magnetoconductance curves were observed in the whole magnetic field region. When $V_g \geq -20$ V, the magnetoconductance exhibits negative magnetoconductance at low magnetic field, which is the characteristic feature of the weak anti-localization effect originating from the interference of self-crossing electronic waves in the presence of the spin-orbit interaction. To evaluate the strength of the Rashba spin-orbit interaction at various V_g, we analyzed the observed magnetoconductance curves based on the Maekawa–Fukuyama (MF) localization theory as follows[5,6].

$$\frac{\Delta\sigma(\mathrm{B})}{\sigma_0} = \Psi\left(\frac{B}{B_\mathrm{i} + B_\mathrm{so}}\right) + \frac{1}{2\sqrt{1-\gamma^2}}\Psi\left(\frac{B}{B_\mathrm{i} + B_\mathrm{so}(1+\sqrt{1-\gamma^2}}\right)$$

$$-\frac{1}{2\sqrt{1-\gamma^2}}\Psi\left(\frac{B}{B_\mathrm{i} + B_\mathrm{so}(1-\sqrt{1-\gamma^2}}\right). \quad (1)$$

Here, the function Ψ is defined as $\Psi(x) = \ln(x) + \psi(\frac{1}{2} + \frac{1}{x})$, where $\psi(x)$ is the digamma function and $\sigma_0 = \mathrm{e}^2/\pi h$ (e is the electron charge and h is Planck constant) is universal value of conductance. $B_\mathrm{i} = \hbar/4\mathrm{e}D\tau_\mathrm{i}$, $B_\mathrm{so} = \hbar/4\mathrm{e}D\tau_\mathrm{so}$, and $\gamma = g\mu_\mathrm{B}B/4\mathrm{e}DB_\mathrm{so}$ are parameters indicating inelastic scattering field, spin-orbit interaction field, and Zeeman correction term, respectively, where D is the diffusion constant, τ_i is the inelastic scattering time, τ_so is the spin relaxation time, g is electrons g factor and μ_B is the Bohr magneton. In the $LaAlO_3/SrTiO_3$ interface, the spin relaxation time τ_so is dominated by D'yakonov-Perel' (DP) mechanism[2,7]. In this scenario, the

spin relaxation time τ_{so} and the Rashba spin-orbit interaction constant α are related through $\tau_{\mathrm{so}} = \hbar^4/4\alpha^2 m^2 2D$, where $\hbar$ is the reduced Planck constant and m is the carrier mass.

Supplementary Fig. 6 displays the best fitting results according to equation (1), which allows us to trace the V_{g} dependence of the parameters $B_{\mathrm{i,so}}$ and γ. To extract relaxation times $\tau_{\mathrm{i,so}}$ from these parameters, we need to determine the V_{g} dependence of the diffusion coefficient. For this purpose, we measured the V_{g} dependence of the sheet carrier concentration n_{s} by using Hall effect measurements (Supplementary Fig. 7d). An estimate of the Fermi velocity v_{F} and the elastic scattering time using a parabolic dispersion relation with a fixed effective mass $m^* = 2m_{\mathrm{e}}$ (m_{e} is the bare electron mass) enable us to evaluate the diffusion coefficient $D = \frac{v_{\mathrm{F}}^2\tau}{2}$. When $V_{\mathrm{g}} = -40$ V, the obtained inelastic scattering time is shorter than the spin relaxation time. In this regime, the quantum correction to the conductivity can be ascribed to weak localization because the effect of the spin-orbit interaction is weak compared with the orbital effect of the magnetic field. With increasing V_{g}, the spin relaxation time significantly decreases by more than 2 orders of magnitude, whereas the inelastic scattering time remains relatively constant (Supplementary Fig. 7c). Thus, a weak anti-localization characterized by a strong spin-orbit interaction was observed with increasing V_{g}. The estimated spin-orbit coupling constant α and spin splitting energy Δ were substantially enhanced across the quantum critical point (Supplementary Fig. 7a). The spin-orbit interaction significantly increases in between $V_g = +10$ V and $V_g = +20$ V (acrossing Lifshitz point), while crossover between weak localization and weak anti-localization occurs in between $V_g = -40$ V and $V_g = -20$ V. This discrepancy could be attributed to the fact that the crossover between weak localization and weak anti-localization is associated with relative scale between phase coherence

length and spin diffusion length. The estimated spin-orbit constant increases with increasing V_g even below Lifshitz point (Supplementary Fig. 7a). Thus, the change of relative scale between phase coherence length and spin diffusion length may occur before the Lifshitz transition as evidenced in Supplementary Fig. 7c.

In Supplementary Fig. 8, we displayed the estimated effective mass m^* obtained with the assumption of DP spin relaxation, where the spin-orbit scattering time (τ_{so}) follows $\frac{2\pi}{\tau_{\text{so}}} = \Omega_{\text{so}}^2 \tau$. $\Delta_{\text{so}} \equiv \hbar\Omega_{\text{so}} = 2\alpha k_{\text{F}}$ is the Rashba energy (k_{F} is the Fermi momentum). Then, the effective mass can be expressed as follows[8]

$$m^* = \frac{h^2}{4\pi\alpha}\sqrt{\frac{B_{\text{so}}}{\Phi_0}} \tag{2}$$

where, $\Phi_0 = \frac{h}{2\text{e}}$. If we assume that the Rashba spin-orbit interaction has a linear dependence on the V_g, *i.e.* $\alpha = \lambda E$, in wedge model (λ is the material specific Rashba spin-orbit coefficient, E is the interfacial electric field along the normal direction of the interface)[8], we could estimate V_g dependence of m^*. The variation of effective mass upon increasing V_g reflects the charge transport evolving from being d_{xy} dominated ($m^* \sim 0.62 m_{\text{e}}$) to being d_{xz}, d_{yz} dominated ($m^* \sim 2.3 m_{\text{e}}$) across the Lifshitz transition, in consistent with the previous report[8].

References

1 Brinkman, A. et al. Magnetic effects at the interface between non-magnetic oxides. *Nat. Mater.* **6**, 493-496 (2007).

2 Caviglia, A. D. et al. Tunable rashba spin-orbit interaction at oxide interfaces. *Phys. Rev. Lett.* **104**, 126803 (2010).

3 Ayino, Y. et al. Ferromagnetism and spin-dependent transport at a complex oxide interface. *Phys. Rev. Mater.* **2**, 031401(R) (2018).

4 Avci, C. O. et al. Unidirectional spin Hall magnetoresistance in ferromagnet/normal metal bilayers. *Nat. Phys.* **11**, 570-575 (2015).

5 Maekawa, S. & Fukuyama, H. Magnetoresistance in Two-Dimensional Disordered-Systems - Effects of Zeeman Splitting and Spin-Orbit Scattering. *J. Phys. Soc. Jpn.* **50**, 2516-2524 (1981).

6 Knap, W. et al. Weak antilocalization and spin precession in quantum wells. *Phys. Rev. B* **53**, 3912-3924 (1996).

7 Jin, M. J. et al. Nonlocal Spin Diffusion Driven by Giant Spin Hall Effect at Oxide Heterointerfaces. *Nano Lett.* **17**, 36-43 (2017).

8 Fete, A., Gariglio, S., Caviglia, A. D., Triscone, J. M. & Gabay, M. Rashba induced magnetoconductance oscillations in the $LaAlO_3$-$SrTiO_3$ heterostructure. *Phys. Rev. B* **86**, 201105(R) (2012).
